# Prognostic Value of Serum and Bronchoalveolar Lavage Fluid Galactomannan Levels in Invasive Aspergillosis: An 8-Year Experience at a Tertiary Cancer Center

**DOI:** 10.3390/jof11050355

**Published:** 2025-05-03

**Authors:** Saliba Wehbe, Anne-Marie Chaftari, Ray Hachem, Hiba Dagher, Andrea Haddad, Ann Philip, Ying Jiang, Ramia Zakhour, Peter Bakht, Jishna Shrestha, Peter Lamie, Robin Sherchan, Jennifer Makhoul, Patrick Chaftari, Issam I. Raad

**Affiliations:** 1 Department of Infectious Diseases, Infection Control and Employee Health, The University of Texas MD Anderson Cancer Center, Houston, TX 77030, USA; achaftari@mdanderson.org (A.-M.C.); rhachem@mdanderson.org (R.H.); hrdagher@mdanderson.org (H.D.); ahaddad@metrohealth.org (A.H.); apphilip@mdanderson.org (A.P.); yijiang@mdanderson.org (Y.J.); pbakht@mdanderson.org (P.B.); jshrestha@mdanderson.org (J.S.); pmlamie@mdanderson.org (P.L.); sherchan.robin@gmail.com (R.S.); jmakhoul@mdanderson.org (J.M.); iraad@mdanderson.org (I.I.R.); 2 Division of Infectious Diseases, Department of Pediatrics, McGovern Medical School at UTHealth, Houston, TX 77030, USA; rzakhour@mdanderson.org; 3 Department of Emergency Medicine, The University of Texas MD Anderson Cancer Center, Houston, TX 77030, USA; pchaftari@mdanderson.org

**Keywords:** galactomannan, invasive aspergillosis

## Abstract

**Background:** Invasive aspergillosis (IA) is a life-threatening fungal infection that primarily affects immunocompromised individuals and has high morbidity and mortality rates, necessitating timely diagnosis and treatment. This study aimed to evaluate the prognostic utility of serum and bronchoalveolar lavage (BAL) fluid galactomannan levels, as well as galactomannan kinetics, in patients with IA. **Methods**: We retrospectively reviewed the medical records of patients who were diagnosed with proven or probable IA from March 2016 to April 2024 at a tertiary cancer center. The collected data included patient characteristics, baseline and peak galactomannan levels in serum and BAL fluid, galactomannan trends, and clinical outcomes. Subgroup analyses were performed to assess the prognostic value of dual-source galactomannan positivity (positive serum and BAL fluid galactomannan levels). **Results:** Elevated baseline serum galactomannan levels independently predicted treatment non-response (*p* = 0.039) and 12-week all-cause mortality (*p* < 0.001). Peak serum and BAL fluid galactomannan levels were strongly associated with poor clinical outcomes (*p* < 0.01). Compared to single-source galactomannan positivity, dual-source galactomannan positivity was linked to reduced treatment response (22% vs. 43%, *p* = 0.01) and higher IA-attributable mortality (52% vs. 27%, *p* = 0.002). Patients with neutropenia had poorer outcomes compared to patients without neutropenia, but neutrophil recovery dramatically improved survival (25% vs. 69% mortality, *p* < 0.0001). Early galactomannan kinetics and malignancy type had limited prognostic value. **Conclusions:** Our findings highlight the potential role of galactomannan as a key biomarker for early prognostication for IA. The strong association between galactomannan levels and clinical outcomes suggests its utility in identifying high-risk patients who may benefit from more aggressive management. Further studies are needed to introduce a nuanced and context-specific use of galactomannan into clinical practice and assess its role as a prognostic biomarker.

## 1. Introduction

Invasive aspergillosis (IA) is a life-threatening fungal infection that predominantly affects immunocompromised individuals, including patients undergoing chemotherapy, hematopoietic stem cell transplant, or solid organ transplant [1,2]. The high morbidity and mortality rates associated with IA demand prompt and precise diagnostic measures to facilitate timely therapeutic intervention [3].

Among the diagnostic tools available, galactomannan, a polysaccharide component of the *Aspergillus* cell wall released during fungal growth, has emerged as a critical biomarker for both the diagnosis and the monitoring of IA. The galactomannan assay, particularly the enzyme immunoassay, detects galactomannan in biological fluids, such as serum and bronchoalveolar lavage (BAL) fluid, providing essential information on the presence of IA [4]. The test provides rapid results, often days earlier than traditional culture methods, which makes it an indispensable tool in clinical settings where timely diagnosis is paramount, such as intensive care units, and for immunocompromised populations [5,6].

While the galactomannan assay has proven indispensable, its utility is not without limitations. For example, its sensitivity can vary significantly depending on the patient population, the type of specimen tested, and the anti-mold treatment, highlighting the need for a nuanced and context-specific approach to the assay’s application [4,7]. The prognostic value of the galactomannan levels is being increasingly recognized, with studies indicating that elevated galactomannan concentrations are correlated with worse clinical outcomes [8]. Furthermore, the kinetics of the galactomannan levels during treatment provide valuable prognostic information. Rising galactomannan levels during therapy are often associated with treatment failure and higher mortality rates, whereas a decrease in the galactomannan levels typically indicates a favorable response to therapy [9,10]. These fluctuations can inform clinicians about disease progression and treatment efficacy, underscoring the importance of regular monitoring the galactomannan levels when managing IA.

Despite significant advances in galactomannan testing, critical gaps persist in understanding the comparative prognostic values of serum and BAL fluid galactomannan levels. Clarifying the relationship between galactomannan positivity in these sources, particularly the implications of dual-source positivity (i.e., concurrent positive serum and BAL fluid galactomannan levels) could potentially enhance diagnostic accuracy and refine prognostic stratification. Additionally, the clinical utility of peak versus baseline galactomannan values remains underexplored.

In the current study, we explored the prognostic utility of serum and BAL fluid galactomannan levels in a cohort of immunocompromised patients who were diagnosed with IA at a tertiary cancer center over an 8-year period. To this end, we evaluated baseline and peak galactomannan values, as well as galactomannan kinetics. Additionally, we examined how factors such as neutropenia, neutropenia recovery, and underlying malignancy type influence prognosis.

## 2. Materials and Methods

### 2.1. Study Design and Data Collection

We retrospectively reviewed the medical records of patients diagnosed with proven or probable IA based on the European Organization for Research and Treatment of Cancer and Mycoses Study Group (EORTC/MSG) criteria [11] at The University of Texas MD Anderson Cancer Center between March 2016 and April 2024. To maintain population homogeneity, patients diagnosed solely based on positive culture results without a corresponding positive baseline galactomannan value were excluded. The data extracted from electronic medical records included patient demographics and characteristics, as well as serum and BAL fluid galactomannan levels from baseline to 12 weeks post-diagnosis. Clinical outcomes were also recorded and comprised therapeutic response at the end of therapy, all-cause mortality, and mortality attributable to invasive aspergillosis (IA) at 6 and 12 weeks post-diagnosis. IA-attributable mortality was defined as death occurring in the absence of clinical improvement and with no alternative cause identified. Therapeutic response was evaluated by a multidisciplinary research team using standardized criteria, incorporating clinical, radiological, and microbiological assessments to ensure objectivity and consistency.

Neutropenia was defined as an absolute neutrophil count (ANC) of ≤500 cells/μL, and recovery was considered achieved when the ANC remained above this threshold for at least seven consecutive days during the course of the infection. BAL fluid samples were collected in sterile saline and tested either directly (neat) or using the supernatant obtained after centrifugation at 10,000 rpm for 10 min. All galactomannan testing was performed using the Platelia™ Aspergillus enzyme immunoassay (EIA) from Bio-Rad Laboratories, Hercules, CA, USA.

### 2.2. Analysis and Outcomes

To assess the prognostic value of serum galactomannan kinetics, we analyzed changes in the galactomannan levels from baseline to the next available value, which was usually obtained within 3 to 14 days after diagnosis (Table S1). Additionally, we conducted a subgroup analysis of the patients who had both serum and BAL fluid galactomannan levels assessed within 1 week of diagnosis. This subgroup was stratified based on galactomannan positivity in serum alone, BAL fluid alone, or both (i.e., dual-source positivity) to compare therapeutic responses and clinical outcomes.

The patient data were securely stored in password-protected electronic case report forms using REDCap version 15.3.1, and all data were de-identified to ensure confidentiality. The study was approved by the institutional review board at MD Anderson, and informed consent was waived due to the retrospective nature of the research.

### 2.3. Statistical Analysis

The Wilcoxon rank-sum test was used to compare continuous variables due to the data not following a normal distribution. Chi-square or Fisher’s exact test was used to compare categorical variables, as appropriate. All tests were two-sided, with a significance level set at 0.05. Statistical analyses were performed using SAS version 9.4 (SAS Institute Inc., Cary, NC, USA).

## 3. Results

The study included 457 immunocompromised patients diagnosed with invasive aspergillosis, with a median age of 59 years (IQR 44–69) and a slight male predominance (59%). Probable IA was identified in 95% of the cases. The majority had hematologic malignancies, most commonly acute myeloid leukemia (45%), and 94% had active cancer at the time of diagnosis. Almost half of the patients (46%) required intensive care unit admission, 30% needed mechanical ventilation, and 52% died within 12 weeks of IA diagnosis from any cause, with 34% of the deaths directly attributable to IA. The baseline galactomannan levels in the cohort showed median BAL fluid and serum values of 2.08 (IQR 1.23–5.37) and 1.84 (IQR 0.98–4.16), respectively.

Patients who did not respond to antifungal therapy at the end of therapy had significantly higher median galactomannan peak values in BAL fluid and in serum than those who responded (BAL, *p* = 0.003; serum, *p* = 0.006) (Table 1). Similar results were observed for patients with 6- and 12-week all-cause mortality who had higher median galactomannan values compared to those who survived (BAL, *p* = 0.007; serum, *p* = 0.003) (Table 1). Baseline serum, but not BAL fluid, galactomannan levels independently predicted non-response to therapy (*p* = 0.039) and 12-week all-cause (*p* < 0.001) and IA-attributable (*p* = 0.005) mortality rates (Table 2).

Compared to patients without neutropenia, patients with neutropenia had reduced treatment response (29% vs. 48%, *p* < 0.001) and higher 12-week IA-attributable mortality (40% vs. 27%, *p* = 0.004) as well as 12-week all-cause mortality (*p* = 0.01) (Table 3). Recovery from neutropenia, however, dramatically altered the outcomes: patients who had neutrophil recovery had significantly reduced 6-week and 12-week all-cause as well as IA-attributable mortality rates (*p* < 0.0001) and were significantly more likely to have a treatment response (60% vs. 18%, *p* < 0.0001) (Table 3). The outcomes did not differ by malignancy type, with patients with hematologic malignancies and solid tumors showing similar mortality rates and treatment responses (Table 4).

Changes in serum galactomannan levels from baseline to follow-up (3 to 14 days after IA diagnosis) were not associated with treatment response or mortality (Table 5). Although the patients who died by 12 weeks of IA diagnosis, either from any cause or from IA-attributable causes, had a smaller decrease in the galactomannan levels compared to the patients who did not die, the trends were not statistically significant (*p* = 0.09 for all-cause mortality; *p* = 0.13 for IFI-attributable mortality).

Dual-source galactomannan positivity (i.e., concurrent positive BAL fluid and serum galactomannan levels) emerged as a particularly potent prognostic indicator. Patients with dual-source positivity had nearly half the treatment response rate (22% vs. 43%, *p* = 0.01) and a nearly two-fold increase in the 12-week IA-attributable mortality rate (52% vs. 27%, *p* = 0.002) compared to patients with single-source positivity, despite similar rates of intensive care unit admission and mechanical ventilation (Table 6).

## 4. Discussion

Our study provides critical insight into prognostic factors for IA in immunocompromised patients, highlighting the roles of both serum and BAL fluid galactomannan levels, the significance of dual-source galactomannan positivity, and the impact of neutropenia and malignancy type. We found that elevated galactomannan levels, whether in serum or in BAL fluid, were strongly associated with treatment failure and increased mortality, aligning with prior research [12,13]. Consistent with our findings, Dabas et al. demonstrated that a serum galactomannan level ≥1.24 is the best predictor of mortality for patients in the intensive care unit [14], and BAL fluid galactomannan levels >2.0 have been shown to have significant prognostic value [13]. Notably, our analysis differentiated between peak and baseline galactomannan values, revealing that peak serum and BAL fluid galactomannan levels were predictive of outcomes, whereas only baseline serum, not BAL fluid, elevated galactomannan levels independently predicted treatment non-response and mortality. This divergence highlights the complementary roles of these biomarkers: BAL fluid galactomannan is highly sensitive for diagnosing localized disease, often outperforming serum galactomannan in this regard [15], and serum galactomannan may better reflect systemic disease burden. Together, these findings suggest that integrating both serum and BAL fluid galactomannan testing into IA disease management could improve risk stratification and therapeutic strategies for IA.

Neutropenia and its recovery were key determinants of outcomes in our cohort and had a significant impact on IA progression. Patients with neutropenia had significantly lower treatment response rates and higher IA-attributable mortality rates, which is consistent with previous studies that highlight neutropenia as a major risk factor for IA progression [16]. Importantly, recovery from neutropenia dramatically improved the outcomes, with a significant reduction in 12-week mortality and a threefold increase in treatment response. These findings align with research showing that neutrophil recovery is associated with better outcomes for patients with IA [17,18], suggesting that interventions aimed at promoting hematopoietic reconstitution could have a noteworthy impact on survival.

Interestingly, the outcomes did not differ by malignancy type, with patients with hematologic malignancies and solid tumors having similar mortality and treatment response rates. This finding contrasts with those of some studies that suggest that patients with solid tumors may have better outcomes due to less profound immunosuppression [19,20]. Our findings suggest that IA severity in immunocompromised patients may be driven more by fungal burden and immune status than by the specific underlying malignancy. This finding has important implications for risk stratification, as it highlights the need to focus on biomarkers of fungal burden and immune recovery rather than on cancer type alone.

The dynamics of the changes in the galactomannan levels during early treatment demonstrated limited prognostic utility in our analysis. Although patients who died by 12 weeks after IA diagnosis had smaller declines in the galactomannan levels compared to patients who did not die, this trend was weak and lacked statistical significance. This observation contrasts with the findings of prior studies that identified galactomannan clearance during treatment as a predictor of survival [21,22]. The limited predictive value of early galactomannan changes may reflect the complex interactions among host factors, antifungal therapy, and fungal burden.

In our study, dual-source galactomannan positivity (i.e., elevated galactomannan levels in both BAL fluid and serum) emerged as a robust prognostic indicator, with patients who had elevated galactomannan levels in both samples experiencing significantly worse outcomes. This dual-source positivity likely indicates a more disseminated or refractory disease state, suggesting that dual-source positivity could help identify high-risk patients. While previous studies have primarily examined the prognostic value of serum or BAL fluid galactomannan separately, our findings highlight the potential of dual-source galactomannan positivity to guide therapeutic escalation and improve outcomes for critically ill patients with IA.

While our study provides valuable insights, it has several limitations. First, its retrospective design limits our ability to establish causal relationships. The cohort primarily consisted of patients with hematologic malignancies treated at a single oncohematology center, which may restrict the generalizability of the findings to other immunocompromised populations or clinical settings. Additionally, the timing of the serial galactomannan measurements was inconsistent, potentially influencing the interpretation of galactomannan dynamics. Lastly, variations in antifungal treatment regimens were not accounted for, which could impact the outcomes. Future prospective studies with standardized protocols are needed to validate these findings and refine prognostic models.

## 5. Conclusions

Our study underscores the critical roles of serum and BAL fluid galactomannan levels, dual-source galactomannan positivity, and neutropenia in predicting outcomes of IA in immunocompromised patients. Peak galactomannan levels, serum baseline galactomannan levels, and dual-source galactomannan positivity were strongly associated with poor outcomes, and neutrophil recovery significantly improved survival. These findings highlight the importance of integrating a refined galactomannan testing protocol into risk stratification and therapeutic decision-making for high-risk patients.

## Figures and Tables

**Table 1:** Peak GM values in patients with different outcomes and GM sources.

Outcome	BAL Source			Serum Source		
	N	Highest GM Value, Median (IQR)	*p* Value	N	Highest GM Value, Median (IQR)	*p* Value
No ICU admission	97	2.64 (1.34–6.26)	0.85	111	3.60 (1.46–7.18)	0.74
ICU admission	67	2.17 (1.36–7.01)		111	3.71 (1.63–7.20)	
No mechanical ventilation	117	2.52 (1.36–6.26)	0.81	153	3.69 (1.60–7.22)	0.47
Mechanical ventilation	47	2.56 (1.35–7.02)		69	3.16 (1.56–7.01)	
No response at EOT	88	3.33 (1.58–7.29)	0.003	137	4.91 (1.69–7.27)	0.006
Response at EOT	58	1.72 (1.18–3.87)		68	2.48 (1.24–5.81)	
No death of any cause at 6 weeks	108	1.87 (1.23–4.84)	0.005	130	2.87 (1.28–6.69)	0.009
Death of any cause at 6 weeks	54	4.58 (1.80–7.24)		90	5.34 (1.98–7.29)	
No IA-attributable death at 6 weeks	122	1.97 (1.24–5.37)	0.019	152	2.87 (1.45–6.98)	0.006
IA-attributable death at 6 weeks	35	5.01 (1.71–7.33)		59	5.48 (2.50–7.72)	
No death of any cause at 12 weeks	89	1.83 (1.22–4.37)	0.007	95	2.66 (1.27–6.25)	0.003
Death of any cause at 12 weeks	73	3.45 (1.61–7.18)		125	5.14 (1.86–7.50)	
No IA-attributable death at 12 weeks	108	1.90 (1.26–5.35)	0.021	127	2.66 (1.40–6.35)	0.001
IA-attributable death at 12 weeks	41	3.97 (1.71–7.25)		79	5.48 (2.10–7.61)	

**Abbreviations:** GM, galactomannan; IQR, interquartile range; BAL, bronchoalveolar lavage; ICU, intensive care unit; EOT, end of therapy; IA, Invasive Aspergillosis.

**Table 2:** Baseline GM values in patients with different outcomes and GM sources.

Outcome	BAL Source			Serum Source		
	N	Baseline GM Value, Median (IQR)	*p* Value	N	Baseline GM Value, Median (IQR)	*p* Value
No ICU admission	123	2.63 (1.36–6.26)	0.051	121	1.54 (0.94–3.45)	0.13
ICU admission	84	1.73 (1.14–4.41)		123	2.07 (1.06–4.87)	
No mechanical ventilation	147	2.52 (1.36–6.26)	0.17	168	1.68 (0.98–3.62)	0.36
Mechanical ventilation	60	1.85 (1.14–4.41)		76	2.09 (1.03–5.13)	
No response at EOT	107	2.53 (1.35–6.45)	0.20	150	1.95 (1.03–4.61)	0.039
Response at EOT	77	1.87 (1.20–3.88)		73	1.48 (0.79–3.04)	
No death of any cause at 6 weeks	137	1.83 (1.20–4.37)	0.062	141	1.46 (0.90–2.97)	<0.001
Death of any cause at 6 weeks	67	2.63 (1.55–6.45)		101	2.50 (1.13–5.64)	
No IA attributable death at 6 weeks	154	1.89 (1.20–5.14)	0.24	164	1.56 (0.93–3.06)	0.003
IA-attributable death at 6 weeks	42	2.60 (1.36–6.88)		69	3.18 (1.16–6.24)	
No death of any cause at 12 weeks	112	1.85 (1.19–4.84)	0.29	102	1.45 (0.89–2.59)	<0.001
Death of any cause at 12 weeks	92	2.53 (1.36–6.10)		140	2.26 (1.10- 5.44)	
No IA-attributable death at 12 weeks	135	1.90 (1.23–6.02)	0.53	136	1.54 (0.91–2.69)	0.005
IA-attributable death at 12 weeks	50	2.60 (1.35–6.30)		91	2.63 (1.10–5.64)	

**Abbreviations:** GM, galactomannan; IQR, interquartile range; BAL, bronchoalveolar lavage; ICU, intensive care unit; EOT, end of therapy; IA, Invasive Aspergillosis.

**Table 3:** Outcomes of patients with and without neutropenia and the effect of neutrophil recovery.

Outcome	Non-Neutropenia (*n*= 196)	Neutropenia (*n* = 258)	*p* Value	No Neutrophil Recovery (*n* = 186)	Neutrophil Recovery (*n*= 72)	*p* Value
ICU admission	81/195 (42)	126/257 (49)	0.11	98/185 (53)	28 (39)	0.043
Mechanical ventilation	65 (33)	71/256 (28)	0.21	58/184 (32)	13 (18)	0.031
Response at EOT	83/174 (48)	68/234 (29)	<0.001	30/171 (18)	38/63 (60)	<0.0001
6-week all-cause mortality	71/192 (37)	97/256 (38)	0.84	92/185 (50)	5/71 (7)	<0.0001
6-week IA-attributable mortality	44/187 (24)	68/244 (28)	0.31	65/173 (38)	3/71 (4)	<0.0001
12-week all-cause mortality	86/192 (45)	146/256 (57)	0.01	128/185 (69)	18/71 (25)	<0.0001
12-week IA-attributable mortality	48/180 (27)	94/234 (40)	0.004	85/166 (51)	9/68 (13)	<0.0001

**Abbreviations:** ICU, intensive care unit; EOT, end of therapy; IA, Invasive Aspergillosis.

**Table 4:** Outcomes of patients according to malignancy type.

Outcome	Hematologic Malignancy (*n*= 418)	Solid Tumor (*n*= 33)	*p* Value
ICU admission	195/417 (47)	11 (33)	0.14
Mechanical ventilation	126/417 (30)	9 (27)	0.72
Response at EOT	136/375 (36)	12/30 (40)	0.68
6-Week all-cause mortality	158/412 (38)	12 (36)	0.82
6-Week IA-attributable mortality	107/396 (27)	6/32 (19)	0.31
12-Week all-cause mortality	216/412 (52)	17 (52)	0.92
12-Week IA-attributable mortality	137/382 (36)	6/29 (21)	0.10

**Abbreviations:** ICU, intensive care unit; EOT, end of therapy; IA, Invasive Aspergillosis.

**Table 5:** Changes in serum GM values from baseline to follow-up (3 to 14 days after diagnosis) for different outcomes.

Outcome	*n*	Change in GM Value, Median (IQR)	*p* Value
No ICU admission	58	−0.36 (−1.48–0.25)	0.47
ICU admission	52	−0.13 (−1.30–0.41)	
No mechanical ventilation	80	−0.28 (−1.48–0.31)	0.44
Mechanical ventilation	29	−0.03 (−1.08–0.39)	
No response at EOT	64	−0.13 (−1.06–0.43)	0.29
Response at EOT	36	−0.18 (−1.96–0.27)	
No death of any cause at 6 weeks	65	−0.56 (−1.48–0.17)	0.15
Death of any cause at 6 weeks	45	−0.10 (−1.02–0.45)	
No IA-attributable death at 6 weeks	75	−0.56 (−1.68–0.36)	0.32
IA-attributable death at 6 weeks	27	−0.03 (−1.08–0.37)	
No death of any cause at 12 weeks	49	−0.71 (−1.68–0.11)	0.09
Death of any cause at 12 weeks	61	−0.10 (−1.08–0.45)	
No IA-attributable death at 12 weeks	63	−0.59 (−1.93–0.17)	0.13
IA-attributable death at 12 weeks	34	−0.03 (−1.08–0.43)	

Note: A negative GM value means a decrease in the GM value from baseline to the earliest follow-up test between day 3 and day 14 after baseline, and a positive value means an increase in the GM value from baseline to the earliest follow-up test 3 to 14 days after baseline. **Abbreviations:** GM, galactomannan; IQR, interquartile range; ICU, intensive care unit; EOT, end of therapy; IA, Invasive Aspergillosis.

**Table 6:** Single-source (serum or BAL fluid) and dual-source (serum and BAL fluid) galactomannan positivity and patient outcomes.

Outcome	Dual-Source Positivity (DSP) (*n*= 67) *	Single-Source Positivity (SSP) (*n* = 93)	SSP from Serum	SSP from BAL	*p* Value
ICU admission during infection	32 (48)	40 (43)	20 (50)	20 (50)	0.55
Mechanical ventilation during infection	21/66 (32)	29 (31)	15 (52)	14 (48)	0.93
Response to therapy at EOT	14/63 (22)	37/87 (43)	19 (51)	18 (49)	0.01
6-week all-cause mortality	34 (51)	27/92 (29)	11 (41)	16 (59)	0.006
6-week IA-attributable mortality	23/63 (37)	15/87 (17)	6 (40)	9 (60)	0.007
12-week all-cause mortality	45 (67)	44/92 (48)	22 (50)	22 (50)	0.015
12-week IA-attributable mortality	32/61 (52)	22/82 (27)	11 (50)	11 (50)	0.002

* A positive serum galactomannan test means either a single test with a value ≥1.0 or at least two tests with a value ≥0.5. A positive BAL fluid galactomannan test means a test with a value ≥1.0. **Abbreviations:** BAL, bronchoalveolar lavage; ICU, intensive care unit; EOT, end of therapy; IA, Invasive Aspergillosis.

## Data Availability

The original contributions presented in this study are included in the article. Further inquiries can be directed to the corresponding author.

## Supplementary Materials

The following supporting information can be downloaded at: https://www.mdpi.com/article/10.3390/jof11050355/s1, Table S1: Serum GM values from baseline up to the end of follow-up (3–14 days) after diagnosis.

## References

[1] Morgan J., Wannemuehler K.A., Marr K.A., Hadley S., Kontoyiannis D.P., Walsh T.J., Fridkin S.K., Pappas P.G., Warnock D.W. (2005). Incidence of invasive aspergillosis following hematopoietic stem cell and solid organ transplantation: Interim results of a prospective multicenter surveillance program. Med. Mycol..

[2] Nosari A., Oreste P., Cairoli R., Montillo M., Carrafiello G., Astolfi A., Muti G., Marbello L., Tedeschi A., Magliano E. (2001). Invasive aspergillosis in haematological malignancies: Clinical findings and management for intensive chemotherapy completion. Am. J. Hematol..

[3] Denning D.W. (2024). Global incidence and mortality of severe fungal disease. Lancet Infect. Dis..

[4] Gupta A., Capoor M.R., Shende T., Sharma B., Mohindra R., Suri J.C., Gupta D.K. (2017). Comparative evaluation of galactomannan test with bronchoalveolar lavage and serum for the diagnosis of invasive aspergillosis in patients with hematological malignancies. J. Lab. Physicians.

[5] Maertens J., Van Eldere J., Verhaegen J., Verbeken E., Verschakelen J., Boogaerts M. (2002). Use of Circulating Galactomannan Screening for Early Diagnosis of Invasive Aspergillosis in Allogeneic Stem Cell Transplant Recipients. J. Infect. Dis..

[6] Schroeder M., Simon M., Katchanov J., Wijaya C., Rohde H., Martin C., Laqmani A., Wichmann D., Fuhrmann V., Kluge S. (2016). Does Galactomannan Testing Increase Diagnostic Accuracy for IPA in the ICU? A Prospective Observational Study. Crit. Care.

[7] Ağca H., Ener B., Yılmaz E., Ursavaş A., Kazak E., Özkocaman V., Cetinoglu E.D., Dilektaşlı A.G., Akalın H., Özkalemkaş F. (2013). Comparative Evaluation of Galactomannan Optical Density Indices and Culture Results in Bronchoscopic Specimens Obtained From Neutropenic and Non-neutropenic Patients. Mycoses.

[8] Koehler P., Salmanton-García J., Gräfe S.K., Koehler F.C., Mellinghoff S.C., Seidel D., Steinbach A., Cornely O.A. (2019). Baseline predictors influencing the prognosis of invasive aspergillosis in adults. Mycoses.

[9] Chai L.Y., Kullberg B.J., Earnest A., Johnson E.M., Teerenstra S., Vonk A.G., Schlamm H.T., Herbrecht R., Netea M.G., Troke P.F. (2014). Voriconazole or amphotericin B as primary therapy yields distinct early serum galactomannan trends related to outcomes in invasive aspergillosis. PLoS ONE.

[10] Mercier T., Guldentops E., Lagrou K., Maertens J. (2018). Galactomannan, a Surrogate Marker for Outcome in Invasive Aspergillosis: Finally Coming of Age. Front. Microbiol..

[11] De Pauw B., Walsh T.J., Donnelly J.P., Stevens D.A., Edwards J.E., Calandra T., Pappas P.G., Maertens J., Lortholary O., Kauffman C.A. (2008). Revised Definitions of Invasive Fungal Disease from the European Organization for Research and Treatment of Cancer/Invasive Fungal Infections Cooperative Group and the National Institute of Allergy and Infectious Diseases Mycoses Study Group (EORTC/MSG) Consensus Group. Clin. Infect. Dis..

[12] Woods G., Miceli M.H., Grazziutti M.L., Zhao W., Barlogie B., Anaissie E. (2007). Serum Aspergillus galactomannan antigen values strongly correlate with outcome of invasive aspergillosis: A study of 56 patients with hematologic cancer. Cancer.

[13] Luong M.-L., Filion C., Labbé A.-C., Roy J., Pépin J., Cadrin-Tourigny J., Carignan S., Sheppard D.C., Laverdière M. (2010). Clinical utility and prognostic value of bronchoalveolar lavage galactomannan in patients with hematologic malignancies. Diagn. Microbiol. Infect. Dis..

[14] Dabas Y., Mohan A., Xess I. (2018). Serum galactomannan antigen as a prognostic and diagnostic marker for invasive aspergillosis in heterogeneous medicine ICU patient population. PLoS ONE.

[15] Hsu L.-Y., Ding Y., Phua J., Koh L.-P., Chan D.S., Khoo K.-L., Tambyah P.A. (2010). Galactomannan testing of bronchoalveolar lavage fluid is useful for diagnosis of invasive pulmonary aspergillosis in hematology patients. BMC Infect. Dis..

[16] Cornillet A., Camus C., Nimubona S., Gandemer V., Tattevin P., Belleguic C., Chevrier S., Meunier C., Lebert C., Aupée M. (2006). Comparison of Epidemiological, Clinical, and Biological Features of Invasive Aspergillosis in Neutropenic and Nonneutropenic Patients: A 6-Year Survey. Clin. Infect. Dis..

[17] Kontoyiannis D.P., Selleslag D., Mullane K., Cornely O.A., Hope W., Lortholary O., Croos-Dabrera R., Lademacher C., Engelhardt M., Patterson T.F. (2018). Impact of unresolved neutropenia in patients with neutropenia and invasive aspergillosis: A post hoc analysis of the SECURE trial. J. Antimicrob. Chemother..

[18] Walsh T.J., Anaissie E.J., Denning D.W., Herbrecht R., Kontoyiannis D.P., Marr K.A., Morrison V.A., Segal B.H., Steinbach W.J., Stevens D.A. (2008). Treatment of Aspergillosis: Clinical Practice Guidelines of the Infectious Diseases Society of America. Clin. Infect. Dis..

[19] Dib R.W., Khalil M., Fares J., Hachem R.Y., Jiang Y., Dandachi D., Chaftari A.M., Raad I.I. (2020). Invasive pulmonary aspergillosis: Comparative analysis in cancer patients with underlying haematologic malignancies versus solid tumours. J. Hosp. Infect..

[20] Ohmagari N., Raad I.I., Hachem R., Kontoyiannis D.P. (2004). Invasive aspergillosis in patients with solid tumors. Cancer.

[21] Mercier T., Wera J., Chai L.Y.A., Lagrou K., Maertens J. (2020). A Mortality Prediction Rule for Hematology Patients with Invasive Aspergillosis Based on Serum Galactomannan Kinetics. J. Clin. Med..

[22] Bergeron A., Porcher R., Menotti J., Poirot J.L., Chagnon K., Vekhoff A., Cornet M., Isnard F., Raffoux E., Brethon B. (2012). Prospective evaluation of clinical and biological markers to predict the outcome of invasive pulmonary aspergillosis in hematological patients. J. Clin. Microbiol..

