# Supplementary material for: Prognostic Value of Serum and Bronchoalveolar Lavage Fluid Galactomannan Levels in Invasive Aspergillosis: An 8-Year Experience at a Tertiary Cancer Center
Source: J Fungi (Basel). 2025 May 3;11(5):355. doi: 10.3390/jof11050355 (PMC12112564; doi:10.3390/jof11050355)
Supplement: Supplementary file 1 [file jof-11-00355-s001.zip › jof-3543160-supplementary.pdf]

Supplementary Table S1. Serum GM values from baseline up to the end of follow-up (3-14 days) after diagnosis.

Serum GM value	Baseline	Day 3	Day 4	Day 5	Day 6	Day 7	Day 8
Median	1.48	0.53	1.53	0.15	0.40	0.72	0.33
IQR	0.81 - 3.42	0.06 - 1.28	0.09 - 6.24	0.06 - 1.78	0.17 - 1.06	0.28 - 1.69	0.09 - 1.24
Number of patients	211	50	27	31	10	19	24

Serum GM value	Day 9	Day 10	Day 11	Day 12	Day 13	Day 14
Median	1.59	0.16	0.53	5.23	1.78	0.17
IQR	0.82 - 2.86	0.08 - 0.90	0.10 - 6.12	0.32 - 7.34	0.08 - 4.58	0.06 - 0.68
Number of patients	12	13	10	11	4	11

Abbreviations: GM, galactomannan; IQR, interquartile range
